# Combination of Microfluidic Loop-Mediated Isothermal Amplification with Gold Nanoparticles for Rapid Detection of *Salmonella* spp. in Food Samples

**DOI:** 10.3389/fmicb.2017.02159

**Published:** 2017-11-06

**Authors:** Alejandro Garrido-Maestu, Sarah Azinheiro, Joana Carvalho, Sara Abalde-Cela, Enrique Carbó-Argibay, Lorena Diéguez, Marek Piotrowski, Yury V. Kolen’ko, Marta Prado

**Affiliations:** International Iberian Nanotechnology Laboratory, Braga, Portugal

**Keywords:** microfluidics, gold nanoparticles, *Salmonella* spp., LAMP, *invA*

## Abstract

Foodborne diseases are an important cause of morbidity and mortality. According to the World Health Organization, there are 31 main global hazards, which caused in 2010 600 million foodborne illnesses and 420000 deaths. Among them, *Salmonella* spp. is one of the most important human pathogens, accounting for more than 90000 cases in Europe and even more in the United States per year. In the current study we report the development, and thorough evaluation in food samples, of a microfluidic system combining loop-mediated isothermal amplification with gold nanoparticles (AuNPs). This system is intended for low-cost, *in situ*, detection of different pathogens, as the proposed methodology can be extrapolated to different microorganisms. A very low limit of detection (10 cfu/25 g) was obtained. Furthermore, the evaluation of spiked food samples (chicken, turkey, egg products), completely matched the expected results, as denoted by the index kappa of concordance (value of 1.00). The results obtained for the relative sensitivity, specificity and accuracy were of 100% as well as the positive and negative predictive values.

## Introduction

*Salmonella* spp. continues to be a major health issue, not only in Europe where 94625 cases were reported in 2015, but worldwide. It has been estimated that *Salmonella enterica* causes approximately 1.2 million illnesses and 450 deaths per year in the United States (Carroll et al., 2017; Efsa and Ecdc, 2017). These figures highlight that additional improvement in the current analysis methodologies are still needed.

When talking about bacterial foodborne pathogens, official methods are based on classical microbiology which require several days for bacterial isolation and identification. These methods present the additional drawback of not being able of detecting viable but non-culturable bacteria (VBNC). Fast and reliable analytical methods are needed by both, the industry and control laboratories. The main objectives of such methods are to ensure the health of consumers, to easily determine whether a food product has been contaminated, and if possible, identify how and when this contamination occurred. This is needed in order to establish the proper corrective measures (Prado et al., 2016a). Molecular methods have, now for long, demonstrated capability of overcoming the limitations associated with culture based methods (Garrido-Maestu et al., 2014; Chapela et al., 2015; Valderrama et al., 2016), being polymerase chain reaction (PCR) the most widely used and accepted (Garrido et al., 2013; Ceuppens et al., 2014). Furthermore, there is an increase interest on the development of miniaturized devices that would allow *in situ* and/or economic monitoring of food products. However, miniaturization of PCR instrumentation, although possible and feasible (Jia et al., 2007; Wang et al., 2009; Ha and Lee, 2015), requires the capacity of accurate thermal cycling of the sample, since the speed of temperature transitions and the thermal homogeneity throughout the PCR mixture are essential for the run time, efficiency and specificity of the amplification reactions (Wittwer et al., 1990). Such demands concerning accurate thermal control, limit the number of materials that can be used for the fabrication of miniaturized devices (Jia et al., 2007), and consequently increase fabrication costs. Therefore, novel isothermal amplification techniques have emerged in recent years with the goal of providing an analytical solution to some of the drawbacks associated with PCR/qPCR, specially due to their simplicity and reduced thermal budget (Kaprou et al., 2015). Among them, loop-mediated isothermal amplification (LAMP) has become the most popular technique (Mori and Notomi, 2009; Fang et al., 2010b; Rafati and Gill, 2015).

It has been reported that in LAMP, along with specific DNA amplification, an insoluble by product is formed (Mg_2_P_2_O_7_), what allows to directly determine if a particular reaction is positive or negative by naked-eye observation (Mori et al., 2001). However, several different approaches have been published, what indicates that it may not be as straightforward as expected, and thus requiring for trained analysts (Goto et al., 2009; Wan et al., 2012; Wang et al., 2012; Birmpa et al., 2015; Tanner et al., 2015). An alternative approach relies on the use of gold nanoparticles (AuNPs), which exhibit a characteristic localized surface plasmon resonance absorption band (LSPR) in the visible light region, being dependent on the interparticle distance (aggregation causes a red shift originating a red-to-purple color change) (Wong et al., 2014; Prado et al., 2016b). Wong et al. (2014), described a methodology based on the functionalization of AuNPs with 11-mercaptoundecanoic acid (MUA), which conferred negative charge to the surface of the particles. This, allowed to control aggregation/separation based on the presence/absence of Mg^2+^ in the LAMP reaction buffer (Wong et al., 2014), provided a visible color change and a more objective result assessment than turbidity.

The potential of microfluidics to create an integrated miniaturized and stand-alone laboratory has long been a dream of the community. This capability is particularly pertinent in low-resource settings where functional laboratories are simply not available (Chiu et al., 2017). Microfluidic technology is an enabling technology for Lab-On-a-Chip (LOC) tests as it allows to reduce sample/reagent consumption, integrated components and functions, and high portability and flexibility. One major challenge is that an easy-to-operate chip inevitably requires complicated fluid circuits and even microfabricated valves or pumps. Microfluidics provide a higher surface to volume ratio, a faster rate of mass and heat transfer, and the ability to precisely handle very small volumes of reagents (Foudeh et al., 2012; Sun et al., 2014). Microfluidic systems allow precise control of mixing in reduced sample volumes, integration with sensing elements, and provide the ideal conditions to develop a LOC systems for molecular detection of bacteria.

The aim of the current study was to determine the possibility of combining a miniaturized device for LAMP based DNA amplification, with functionalized AuNPs for naked-eye detection of *Salmonella* spp. in food samples. Additionally, its application in real food samples was also assessed, to determine its applicability in the food industry as a simple, inexpensive and fast analytical approach.

## Materials and Methods

### Sample Contamination

In order to evaluate the performance of the methodology, food samples were artificially contaminated with *S.* Typhimurium CECT 4594, purchased from the Spanish Type Culture Collection. A pure culture was prepared by inoculating 4 mL of Buffered Peptone Water (BPW, Biokar Diagnostics S.A., France) with a single colony, and incubated overnight at 37°C. After incubation, this culture was 10-fold serially diluted in BPW and used for the inoculation of the food samples (1 mL of the corresponding dilution was added to the mixture of 25 g of food sample and 225 mL of diluent), as well as plated on Tryptic Soy Broth (TSB, Biokar Diagnostics S.A., France) with 15 g/L of agar, to get viable bacterial counts. The plates were incubated at 37°C overnight.

### Food Sample Treatment and DNA Extraction

To reach regulatory limits for most foodstuffs, regarding *Salmonella* spp. (absence/25 g, see European Regulation 2073/2005) a pre-enrichment step was included in the protocol. To this end, 25 g of sample were weighted and mixed with 225 mL of BPW. Positive samples were inoculated with 1 mL of the corresponding dilution of *S.* Typhimurium prepared as detailed above, homogenized for 30 s and incubated at 37°C for 18–24 h (negative samples were directly homogenized and placed in the incubator). After incubation, 1 mL was taken for DNA extraction. Chicken was selected as the reference food type for the determination of the Limit of Detection (LoD), and in addition to this, turkey, and omelet prepared with eggs were also included in the evaluation of the method. Food samples were obtained from local suppliers. A detailed list of the samples analyzed is provided in **Table 1**.

**Table 1:** Samples inoculated with one single strain.

Sample	*N*	Inoculum (cfu/25 g)	Result
Chicken breast	10^∗^	<10	+
Chicken breast	10	-	-
Chicken breast	2	<10	+
Chicken breast	1	10^3^–10^4^	+
Chicken breast	2	10^4^–10^5^	+
Chicken breast	1	10^7^–10^8^	+
Turkey	2	-	-
Turkey	1	<10	+
Turkey	2	10–10^2^	+
Turkey	1	10^3^–10^4^	+
Omelet (egg)	2	-	-
Omelet (egg)	2	10^2^–10^3^	+
Omelet (egg)	2	10^7^–10^8^	+

*N is the number of samples. *^∗^*These samples were used for the determination of the LOD.*

DNA extraction was performed as previously described by Garrido-Maestu et al. (2017). Briefly, the aliquot from the enriched matrix was centrifuged at 2,000 rpm for 2 min, to eliminate large food debris. The supernatant was centrifuged at 13,000 rpm for 5 min, the pellet was rinsed with 1 mL of PBS and centrifuged again. The clean pellet was resuspended in 300 μL of 6% Chelex^®^100 (w/v) (Bio-Rad Laboratories, Inc., United States), incubated at 56°C for 15 min at 1,000 rpm in a Thermomixer comfort (Eppendorf AG, Germany), and finally the bacteria were lysed by heating at 99°C for 10 min. After thermal lysis, the samples were centrifuged at 13,000 rpm for 5 min at 4°C, and the supernatant containing the DNA was transferred to a clean tube which was stored at 4°C until analysis.

### Microfluidic Device

The fabrication of the microfluidic device was achieved through the combination of computer-numerical-control (CNC) polymer machining and polydimethylsiloxane (PDMS) replica molding. The CNC mold was designed using AutoCAD software, and fabricated in poly(methyl methacrylate) (PMMA) material using a CNC miller (FlexiCAM Viper 606). The PDMS prepolymer (Sylgard 184 silicone elastomer kit), was prepared by mixing the base and curing agent at a weight ratio of 10: 1, then was poured onto the PMMA mold, placed under vacuum for 15–20 min to remove the air bubbles and cured in the oven at 65°C for 1 h. After cooling down, the PDMS replica was peeled off from the mold and microfluidic channels were irreversibly sealed against a glass slide using oxygen plasma bonding. The final dimensions of the microfluidic device were 4 mm (thickness) × 76 mm (length) × 26 mm (width) and its pattern included a total of 8 capillarity-driven microchannels with a geometry of 40 mm (length) × 800 μm (depth) × 600 μm (width). Each microchannel had one inlet and one outlet, incorporated in the PMMA mold, and a volume capacity of 20 μL.

### Gold Nanoparticle Synthesis and Characterization

For the synthesis of spherical AuNPs the well-known Turkevich method was followed (Turkevich et al., 1951). Briefly, 5 mL of a 1% solution of trisodium citrate (Na_3_C_6_H_5_O_7_, Sigma-Aldrich, St. Louis, MO, United States) was added to a boiling solution of gold chloride (HAuCl_4_⋅_3_H_2_O, Sigma-Aldrich, St. Louis, MO, United States) (95 mL, 0.5 mM) under vigorous magnetic stirring. After 5 min, the color of the solution turned from pale yellow to intense red.

The AuNPs were characterized by UV-Vis spectra using a NanoDrop 2000c (Thermo Fisher Scientific, Inc., Waltham, MA, United States). Additionally, the morphological characterization of the citrate-stabilized nanoparticles was performed by transmission electron microscopy (TEM). The images were acquired in a JEOL JEM-2100 electron microscope, operated at 200 kV. TEM samples were prepared by dropping ca. 12 μL of the nanoparticles dispersion onto a formvar/carbon-coated Cu grid (400 mesh) placed on a filter paper followed by the evaporation of the solvent at room temperature.

### Gold Nanoparticle Functionalization

The functionalization of the AuNP was performed as described by Wong et al. (2014). Briefly, 20 nM AuNPs and 2 mM MUA (freshly prepared in DMSO) were mixed, and incubated for 24 h at room temperature with constant agitation (1400 rpm, Thermomixer comfort).

### *Salmonella* spp. DNA Amplification and Detection by Microfluidic-AuNP

The detection of *Salmonella* spp. was assessed targeting *invA* gene. To this end, the primers designed by Hara-Kudo et al. (2005) were selected: FIP: GACGACTGGTACTGATCGATAGTTTTTCAACGTTTCCTGCGG, BIP: CCGGTGAAATTATCGCCACACAAAACCCACCGCCAGG, F3: GGCGATATTGGTGTTTATGGGG, B3: AACGATAAACTGGACCACGG.

A final reaction volume of 25 μL was prepared with 3 μL template DNA, 2.5 μL of 10X Isothermal Amplification Buffer (New England BioLabs, Inc., Ipswich, MA, United States), 1 M betaine (Sigma-Aldrich, St. Louis, MO, United States), 0.35 mM dNTP mix (Thermo Fisher Scientific, Inc., Waltham, MA, United States), and 8 U *Bst* 2.0 WarmStart^®^ DNA Polymerase (New England BioLabs, Inc., Ipswich, MA, United States). The primer concentration was 700 and 100 nM for FIP/BIP and F3/B3 respectively. Out of the master mix prepared, 20 μL were loaded in one of the microfluidic channels, which was carefully sealed with a glass slide and few clamps to avoid evaporation, and placed in a conventional laboratory incubator (Memmert GmbH, Schwabach, Germany) with the temperature set at 65°C for 1 h.

After incubation, 4 μL of the LAMP product were diluted with sterile milli-Q water (8.5 μL) and mixed with the functionalized AuNP (final concentration 6 nM), making a final volume of 15 μL. The results (positive-red, negative-purple) were directly assessed by naked-eye observation. For comparison purposes, gel electrophoresis was also performed; to do so a 2% agarose gel was prepared (NzyTech, Lisbon, Portugal) in Sodium Borate buffer prepared as previously described (SB, Brody and Kern, 2004). The gel was stained with 4 μL of Midori Green (Nippon Genetics Europe GmbH, Düren, Germany). One μL of the LAMP-AuNP product was mixed with 5 μL of 6X DNA loading dye (Thermo Fisher Scientific, Inc., Waltham, MA, United States), and loaded in the gel. The samples were separated for 20 min at 300 V, and finally visualized in a GelDoc^TM^ EZ Imager (Bio-Rad Laboratories, Inc., United States). Finally, UV-vis was also measured for positive and negative samples in a NanoDrop 2000c.

### Fitness-for-Purpose of the Method

Following the protocol described above, the complete method was evaluated taking into account the LoD, relative sensitivity (SE), specificity (SP) and accuracy (AC), positive and negative predictive values (PPV, NPV) and the kappa index of concordance (*k*), as previously described (Tomas et al., 2009; Anderson et al., 2011; Garrido et al., 2013). To do so, every sample was classified as being in Positive, or Negative, Agreement (PA and NA) and Positive, or Negative, Deviation respect to the expected results (if the samples were, or not, inoculated with the target microorganism). Then the previously mentioned parameters were calculated based on the following formulas:

SE = [PA/(PA + ND)] x 100

SP = [NA/(PD + NA)] x 100

AC = [(PA + NA)/N] x 100;

where “N” number of analyzed samples.

PPV = [PA/(PA + PD)] x 100

NPV = [NA/(NA + ND)] x 100

*k* = 2 x (PA x NA - ND x PD)/ [(PA + PD) x (PD + NA) + (PA + ND) x (ND + NA)].

The LoD was determined to be the lowest, reproducible, detectable concentration. The rest of the parameters were calculated based on the obtained and expected results.

## Results

### Microfluidics

**FIGURE 1:**
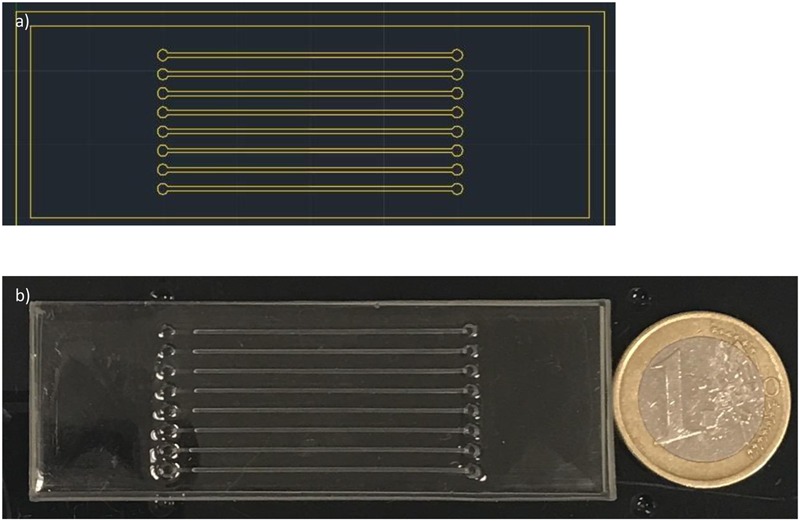
Microfluidic device design and fabrication. **(a)** Schematic microfluidic chip designed with AutoCad. **(b)** Final PDMS microfluidic device (PDMS replica bonded against a glass slide).

The microfluidic device, with eight channels was successfully designed and fabricated as can be observed in **Figures 1a,b**. The capillary-driven chip can perfectly hold 20 μL of reaction mixture in each channel, and was carefully sealed by placing a second glass slide was placed on top, which was held with few clamps. This simple setup avoided evaporation of reagents as well as bubble formation in the channels, when performing the DNA amplification. The enzymatic reaction was not affected by the setup and run as expected.

### AuNP Synthesis and Characterization

**FIGURE 2:**
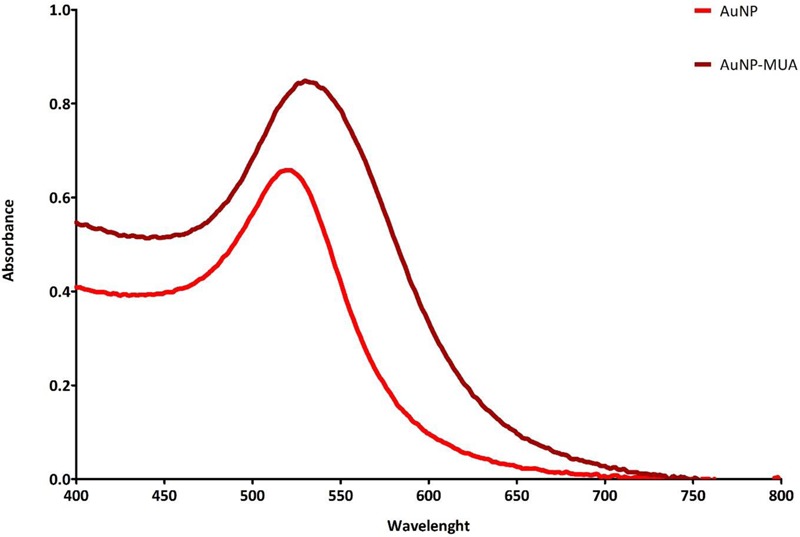
UV-Vis of AuNPs before and after MUA functionalization.

**FIGURE 3:**
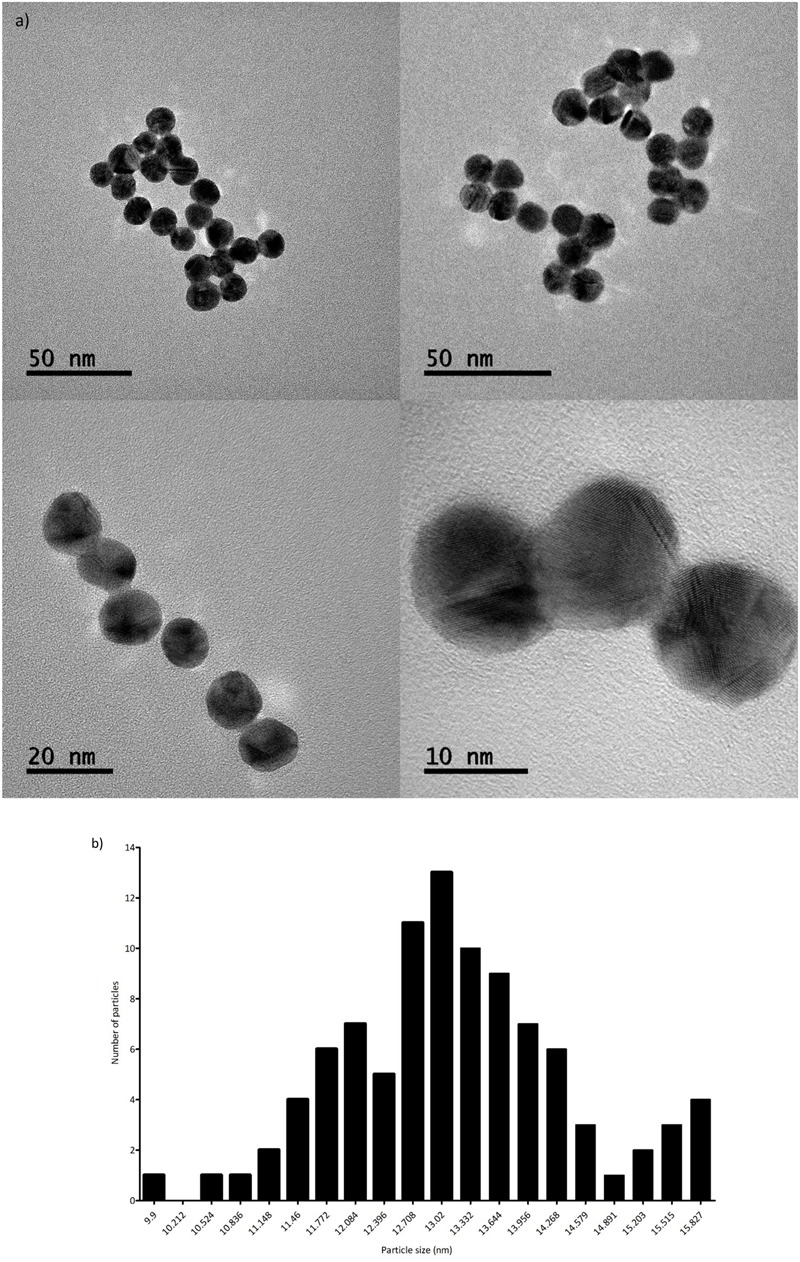
**(a)** AuNP TEM images at different magnifications. **(b)** AuNPs size distribution, based on 96 measurements.

The correct synthesis of AuNPs was confirmed by UV-vis, showing a peak at ≈520 nm, which was still present after MUA functionalization, as depicted in **Figure 2**. In addition to this, the AuNPs were visualized by TEM, confirming spherical morphology and an average size of 13.3 ± 1.2 nm (see **Figures 3a,b**).

### *Salmonella* spp. Detection

**FIGURE 4:**
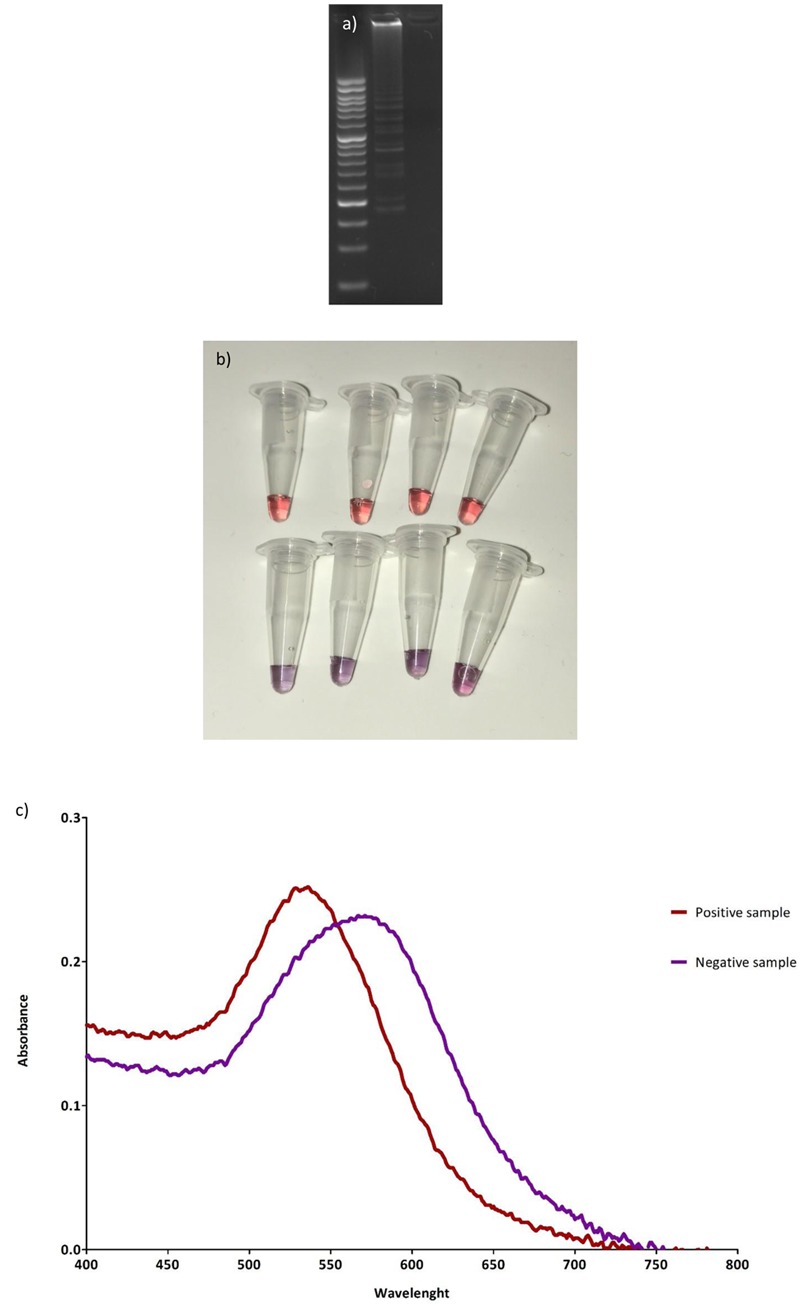
Results of microfluidic-LAMP amplification and MUA-AuNPs detection. **(a)** Gel electrophoresis of a positive and negative sample after LAMP DNA amplification in the microfluidic chip and combination with AuNPs. **(b)** Positive (top) and negative (bottom) samples for *Salmonella* spp. after LAMP DNA amplification in the microfluidic chip, and the addition of AuNPs. **(c)** Typical UV-Vis spectra obtained for positive and negative samples after the addition of AuNPs.

Successful DNA amplification was obtained in the microfluidic chip, as demonstrated by the typical LAMP banding pattern after gel electrophoresis (**Figure 4a**). Clear color differences [red (+) vs. purple (-)] can be observed after combination of functionalized AuNPs with the diluted LAMP amplification product, as shown in **Figure 4b**. These differences are also clearly visualized by UV-Vis, see **Figure 4c**.

### Method Evaluation

The evaluation of the microfluidic-AuNP method demonstrated a LoD of 10 cfu/ 25 g. In addition to this, all performance parameters evaluated (SE, SP, AC, PPV, NPV, and *k*) obtained excellent results when compared to the expected ones (100%), further details are provided in **Table 2**.

**Table 2:** Method evaluation summary.

Bacterium	Gen	N	PA	PD	NA	ND	SE	SP	AC	PPV	NPV	*κ*
*S.* Typhimurium CECT 4594	*invA*	38	24	0	14	0	100	100	100	100	100	1.00

*N, total number of samples; PA, positive agreement; PD, positive deviation; NA, negative agreement; ND, negative deviation; SE, relative sensitivity; SP, relative specificity; AC, relative accuracy; PPV, positive predictive value; NPV, negative predictive value; k, kappa index of concordance, interpretation: 0.61–0.8 substantial agreement; 0.81–1.00 almost complete concordance according to Altman (1991) and Anderson et al. (2011).*

## Discussion

The development and implementation of novel techniques, which allow *in situ*, fast and accurate detection of foodborne pathogens is highly desirable in the food industry. In the current study, the combination of microfluidic-LAMP DNA amplification with AuNP detection was assessed.

The construction of a microfluidic device was successfully accomplished and demonstrated to be suitable for LAMP-based DNA amplification. This approach allowed an increased specificity in bacteria detection, as it was demonstrated in a set of parallel experiments (data not shown). In these experiments, negative samples that were reacted with AuNPs in regular PCR tubes (DNase, RNase and pyrogen free, Nippon Genetics Europe GmbH, Duren, Germany) were determined to be positive; while when the microfluidic chip was used resulted negative, as expected. This may be explained by enhanced performance of the positive reactions due to the advantages of microfluidics previously commented (better heat transfer, controlled mixing and no gravity that prevented NP aggregation, etc.).

Wong et al. (2014) reported for the first time the combination of MUA and AuNPs as a naked-eye approach to asses LAMP DNA amplification. In Wong et al. (2014) study, the functionalized AuNPs were added before the LAMP reaction, what caused particle aggregation. After DNA amplification, in order to see color differences ultrasounds had to be applied to re-disperse the particles in positive samples. This simple treatment, may be problematic when thinking on *in situ* analyses. In the current study, the addition of the AuNPs after DNA amplification, allowed to clearly observe the color changes without applying ultrasounds.

Recently, many studies were published combining LAMP amplification with DNA-functionalized AuNPs (Jaroenram et al., 2012; Arunrut et al., 2013, 2016; Seetang-Nun et al., 2013; Kumvongpin et al., 2016). Even though this approach is expected to present higher specificity, due to the incorporation of the DNA probe, the use of MUA is more economic, the functionalization protocol is easier, and these particles can be incorporated to any LAMP assay, thus expanding their direct applicability (we have tested the sample protocol targeting *S.* Typhimurium, *S.* Enteritidis and *L. monocytogenes* with comparable results in terms of LoD, SE, SP, AC, PPV, NPV and *k*, by just modifying the primers selected, data not shown).

The application of this methodology to *Salmonella*-spiked food samples allowed the reliable detection of the pathogen, even at low concentration (10 cfu/25 g), what is compatible with the needs of the food industry. These results are similar to those obtained with other conventional LAMP methods (Ohtsuka et al., 2005; D’Agostino et al., 2016; Garrido-Maestu et al., 2017), or even with other DNA amplification techniques (González-Escalona et al., 2012; Maurischat et al., 2015; Zhang et al., 2015).

To the best of our knowledge this is the first study combining AuNPs, LAMP and microfluidics, which has undergone an extensive evaluation in food samples (LoD, SE, SP, AC, PPV, NPV, *k*), being critical for future implementation in the food industry. Previous studies have been just focused on the development of the methodology, but a thorough evaluation of its applicability in food products was missing (Fang et al., 2010a,b; Wong et al., 2014; Sayad et al., 2016; Park et al., 2017).

To summarize, in the current study we report the successful combination of microfluidic isothermal DNA amplification with AuNPs-based detection, allowing naked-eye discrimination, for the specific detection of *Salmonella* spp. In food samples. The results obtained indicate the suitability of the methodology for its implementation in the food industry. It is a simple, fast and inexpensive analytical approach for foodborne pathogen detection although an inter-laboratory validation is needed.

## Author Contributions

AG-M designed the experiments and wrote the manuscript. SA performed the DNA amplification experiments and AuNPs evaluation. LD and JC designed the microfluidic system and helped in the optimization of the DNA amplification within the system. SA-C, MP, and YK worked on the synthesis and functionalization of the AuNPs. EC-A and SA-C performed the characterization of the AuNPs by TEM. MP collaborated in the design of the experiments and evaluation of the results. All the co-authors collaborated in the proofreading of the final manuscript.

## Conflict of Interest Statement

The authors declare that the research was conducted in the absence of any commercial or financial relationships that could be construed as a potential conflict of interest.
